# Analysis of copy number variations induced by ultrashort electron beam radiation in human leukocytes in vitro

**DOI:** 10.1186/s13039-019-0433-5

**Published:** 2019-05-16

**Authors:** Tigran Harutyunyan, Galina Hovhannisyan, Anzhela Sargsyan, Bagrat Grigoryan, Ahmed H. Al-Rikabi, Anja Weise, Thomas Liehr, Rouben Aroutiounian

**Affiliations:** 1 Department of Genetics and Cytology, Yerevan State University, 1 Alex Manoogian, 0025 Yerevan, Armenia; 2 CANDLE Synchrotron Research Institute, Acharyan 31, 0040 Yerevan, Armenia; 3 Institute of Human Genetics, Jena University Hospital, Friedrich Schiller University, D-07740 Jena, Germany

**Keywords:** AREAL, Accelerated electrons, Copy number variations (CNVs), Chromosome size, Duplication, Gene density

## Abstract

**Background:**

Environmental risk factors have been shown to alter DNA copy number variations (CNVs). Recently, CNVs have been described to arise after low-dose ionizing radiation in vitro and in vivo. Development of cost- and size-effective laser-driven electron accelerators (LDEAs), capable to deliver high energy beams in pico- or femtosecond durations requires examination of their biological effects. Here we studied in vitro impact of LDEAs radiation on known CNV hotspots in human peripheral blood lymphocytes on single cell level.

**Results:**

Here CNVs in chromosomal regions 1p31.1, 7q11.22, 9q21.3, 10q21.1 and 16q23.1 earlier reported to be sensitive to ionizing radiation were analyzed using molecular cytogenetics. Irradiation of cells with 0.5, 1.5 and 3.0 Gy significantly increased signal intensities in all analyzed chromosomal regions compared to controls. The latter is suggested to be due to radiation-induced duplication or amplification of CNV stretches. As significantly lower gains in mean fluorescence intensities were observed only for chromosomal locus 1p31.1 (after irradiation with 3.0 Gy variant sensitivites of different loci to LDEA is suggested. Negative correlation was found between fluorescence intensities and chromosome size (*r* = − 0.783, *p* < 0.001) in cells exposed to 3.0 Gy irradiation and between fluorescence intensities and gene density (*r* = − 0.475, *p* < 0.05) in cells exposed to 0.5 Gy irradiation.

**Conclusions:**

In this study we demonstrated that irradiation with laser-driven electron bunches can induce molecular-cytogenetically visible CNVs in human blood leukocytes in vitro. These CNVs occur most likely due to duplications or amplification and tend to inversely correlate with chromosome size and gene density. CNVs can last in cell population as stable chromosomal changes for several days after radiation exposure; therefore this endpoint can be used for characterization of genetic effects of accelerated electrons. These findings should be complemented with other studies and implementation of more sophisticated approaches for CNVs analysis.

## Background

Copy number variations (CNVs) that arise due to deletions and duplications in the genome are major contributors to genetic diversity in human population [1]. These changes may lead to phenotypic expression and/or various diseases (cancer, infertility, neurodevelopmental disorders etc.) [2–4], have adaptive effects [5], or can be neutral without significant consequences [1]. Recent studies demonstrated that DNA replication inhibitors have potential to induce CNVs in vitro and in vivo [6–10]. However, natural and artificial environmental factors that may induce CNVs are still poorly studied.

Recent achievements in the field of particle acceleration technologies has led to development of cost- and size-effective laser-driven electron accelerators (LDEAs) [11, 12]. This technology permits to deliver high energy beams (from few MeV up to several hundred MeV) into deep layers of tissue in pico- or femtosecond durations with little lateral spread [13–15]. Regarding the long-term goal to develop and establish laser-based particle accelerators for a future radiotherapeutic treatment of cancer, the radiobiological consequences of laser-driven beams have to be investigated [16].

Different types of DNA damage-associated biomarkers have shown potential as predictors of radiation effects, including cytogenetics (e.g. micronuclei, translocations, dicentrics), proteomics (e.g. g-H2AX, pATM, pP53), genomics (e.g. mRNA, SNPs), or epigenomics (e.g. miRNA, lncRNA) [17]. One of the widely used methods in radiation biology is the comet assay which enables detection of initial radiation-induced DNA breaks [18–20] and analysis of the inter-individual [21] and inter-cellular [22] differences in response to radiation. DNA double-strand breaks (DSBs) represent an important radiation-induced lesion that can be monitored by the gammaH2AX foci analysis [23]. Misrepair of DSBs can produce many types of chromosomal aberrations, in particular DNA rearrangements, including CNVs [9]. While majority of DNA damage can be repaired in several hours or days, chromosomal aberrations are more persistent and even can last up to several years in human. Therefore, evaluation of chromosome damage is a crucial predictor for the degree of radiation induced damage [24–28]. In several studies effects of radiation have been studied focusing on CNVs. In particular, low-dose ionizing radiation was shown to induce de novo CNVs in in vitro studies, with slight prevalence of duplications over deletions. Moreover, hotspots of radiation-induced CNVs have been identified in chromosomal regions 1q44, 3q13.31, 7q11.22, 9p21.3, 10q11.23-q21.1 and 16q23.1 [7]. Furthermore, the frequency of de novo CNVs was significantly elevated in offspring of laboratory mice, exposed to ionizing radiation [9], as well as in the progeny of a human subpopulation accidentally exposed during a radiological accident [10].

Studies of the genetic effects of accelerated particles are still limited. Recently genetic effects of irradiation with LDEA were estimated using comet assay [29, 30], micronucleus test [12] and gammaH2AX foci, reflecting level of DSBs [16, 31, 32]. But for all we know, the effect of accelerated particles on CNVs has not yet been studied. The introduction of additional radiobiological endpoints would greatly expand our understanding of biological effectiveness of laser-driven electron beams.

The efficiency of CNVs as endpoint of genetic effects of ionizing radiation supports the possibility of their application in studies of accelerated electrons. Here we examine in vitro laser-generated ultrashort electron beam irradiation effect on CNVs hotspots in blood lymphocytes of healthy individuals, using parental origin determination fluorescence in situ hybridization (POD-FISH) technique.

## Methods

### Blood cultivation and irradiation

Blood samples were collected by venipuncture from four healthy nonsmoking donors (two female and two male) aged 27–29 years, with normal 46,XX and 46,XY karyotypes, respectively. This study was approved by the Ethic Committee of the National Center of Bioethics (Yerevan State University, Faculty of Biology), and informed consent was obtained from all study donors. The venous blood (2 ml from each donor) was collected into vacutainers with heparin and irradiated by a laser-driven radiofrequency gun-based linear AREAL accelerator (CANDLE, Synchrotron Research Institute, Armenia). Vacutainers with blood samples were placed in the sample holder facing vertically towards the beam coming from the direction of the vacuum window. Earlier the levels of DNA damage in human K-562 cells were studied after irradiation with LDEAs at 0, 2, 4 and 8 Gy at 3.6 and 36 Gy/min dose rates [30]. In current study, relatively low radiation doses and dose rate were selected to avoid pronounced increase of DNA damage. Samples were irradiated with doses of 0.5, 1.5 and 3.0 Gy with a dose rate of 2 Gy/min, the beam charge was 10 pC with the energy of electrons 3 MeV, pulse duration 0.42 fs and pulse repetition rate 2 Hz. After irradiation blood samples were cultivated in RPMI-1640 medium, containing 10% fetal bovine serum, 1% penicillin/streptomycin, and 10 μg/ml phytohemagglutinin-L at 37 °C for 72 h.

### Metaphase chromosome preparation

Metaphase chromosomes were prepared as previously described [33]. Colcemid (0.1 μg/ml final concentration) was added to the culture 1.5 h before harvesting and incubated at 37 °C to achieve metaphase block. At the end of cultivation cells were harvested and centrifuged at 1500 rpm (7 min). The medium was removed completely except for about 0.5 ml of supernatant remaining above the cell pellet. 10 ml of pre-warmed (37 °C) hypotonic solution (0.075 M KCl) was added to the tubes and the contents were mixed gently and incubated for 15 min at 37 °C. After centrifugation and discarding supernatant, cells were fixed in 10 ml of ice-cold fixative (methanol/glacial acetic acid, 3:1 v/v). After incubation 10–15 min at room temperature the cells were centrifuged, supernatant was discarded and 10 ml of fixative was added. After the last centrifugation, cells were resuspended in a small amount of fixative and the suspension was dropped onto a microscope slide, prewashed by fixative. Then the slide was placed on hotplate (51 °C) covered by wet tissue paper and kept until the surface of the slide was dried.

### FISH analysis

POD-FISH has already been successfully used to identify CNVs in human cells [8, 34, 35]. BAC clones for CNV regions were purchased from the Children’s Hospital Oakland Research Institute, Oakland, CA, USA, or kindly provided by the Sanger Centre, UK. BAC DNA was isolated, PCR amplified, and labeled by Nick translation (Roche, Karlsruhe, Germany) [36]. The following BACs were used: RP11-393 N21 for 1p31.1 (TexasRed), RP11-1129E22 for 7q11.22 (SpectrumGreen), RP11-174 K23 for 9q21.3 (TexasRed), RP11-123 L21 for 10q21.1 (SpectrumOrange) and RP11-264 M12 for 16q23.1 (SpectrumGreen). The set of CNV regions was selected on the base of results of genomic distribution of low-dose ionizing radiation-induced CNVs [7]. Image capturing and acquisition were processed with the Isis imaging system (MetaSystems, GmbH, Altlussheim, Germany). For analysis of POD-FISH signals the ImageJ freeware was applied (https://imagej.nih.gov/ij/) [37]. For that purpose images were imported into ImageJ program and size of CNVs was measured on the base of fluorescence intensities of signals from 50 to 60 metaphases for each chromosome region and expressed in arbitrary units (a. u.).

### Statistical analysis

The normality of distribution of FISH signals intensity was analyzed by the Kolmogorov-Smirnov test. Differences between fluorescence intensities of studied chromosome loci of male and female donors were analyzed by Multiple Range test. Significance of difference between untreated and irradiated cells was tested by Student’s *t*-test. Pearson’s correlation was applied for analysis of relation between fluorescence intensities of chromosome loci after irradiation and chromosome size, gene density and interphase position. Statistical analysis was performed using the statistical package Statgraphics Centurion 16.2 and a *p* value < 0.05 was considered statistically significant.

## Results

### Comparison of CNVs in control and irradiated cells

**Fig. 1:**
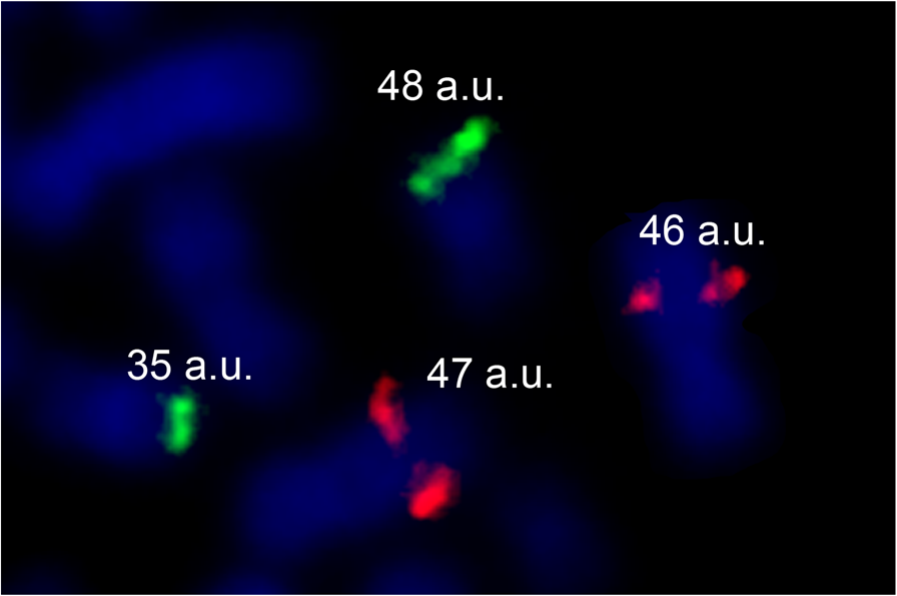
Sample of evaluation of signal intensities by ImageJ program**.** Signal intensities measurements in arbitrary units (a.u.) were done on homologous chromosomes of 9q21.3 (TexasRed) and 16q23.1 (SpectrumGreen) before and after irradiation with accelerated electrons by ImageJ program. Duplication (48 a.u.) was detected as increase of fluorescence intensity of BAC probe for 16q23.1 locus

CNVs of 5 chromosomal regions were analyzed by POD-FISH [8]. Fluorescence intensities of signals reflecting the sizes of the CNVs were compared between treated and untreated samples (Fig. 1). No significant difference in the fluorescence intensities of CNVs was found between males and females; so the pooled data from the four donors are presented in Table 1. Irradiation of cells with 0.5, 1.5 and 3.0 Gy significantly increased signal intensities in all analyzed chromosome regions compared with control due to induced duplications or amplifications. Non-significant increase was shown only in 7q11.22 after irradiation with 1.5 Gy. Studied chromosomal loci demonstrated minor differences in sensitivity to irradiation with LDEA (Table 1). Multiple Range test revealed significantly lower gains in fluorescence intensities in chromosome locus 1p31.1 after irradiation with 3.0 Gy compared to 7q11.22, 9q21.3, 10q21.1 and 16q23.1 loci indicating less LDEA sensitivity of this locus. Significant differences were not observed between loci 7q11.22, 9q21.3, 10q21.1 and 16q23.1 after irradiation with 3.0 Gy, as well as between all studied loci after irradiation with 0.5 and 1.5 Gy.

**Table 1:** Fluorescence intensity of BAC signals (mean ± SD of 50–60 measurements) in different chromosome loci after irradiation

Dose (Gy)	1p31.1	7q11.22	9q21.3	10q21.1	16q23.1
0	57.59 ± 1.75	56.06 ± 4.53	55.13 ± 1.09	56.22 ± 1.71	57.03 ± 1.81
0.5	67.20 ± 1.37*	66.69 ± 2.80*	64.02 ± 2.18*	67.88 ± 3.15*	69.81 ± 0.86*
1.5	64.43 ± 1.79*	63.64 ± 4.40	66.14 ± 2.07*	64.51 ± 4.80*	64.80 ± 0.60*
3.0	64.83 ± 0.94*	69.75 ± 1.37*^a^	69.56 ± 0.91* ^a^	68.86 ± 1.01* ^a^	70.02 ± 2.01*^a^

*p < 0.05—significant difference compared to non-irradiated cells

^a^*p* < 0.05—significantly higher gain in fluorescence intensity compared to 1p31.1 after irradiation with 3.0 Gy

### Correlation of CNVs with chromosome size, gene density and interphase position

To study the involvement of different chromosomes in CNVs instability the Pearson (r) correlations between fluorescence intensities of studied chromosome loci after irradiation with doses 0.5, 1.5 and 3.0 Gy and chromosomes size (bp), gene density (gene/Mb) and interphase position [38, 39] were analyzed (Table 2). Negative correlation was found between fluorescence intensity and chromosome size (*r* = − 0.783, *p* < 0.001) in cells exposed to 3.0 Gy irradiation and between gene density (*r* = − 0.475, *p* < 0.05) in cells exposed to 0.5 Gy irradiation. Statistically significant correlation between fluorescence intensity in irradiated cells and 3D localization of chromosomes in the nucleus was not revealed (*p* > 0.05).

**Table 2:** Correlations of fluorescence intensities in CNVs loci with chromosome size, gene density and interphase position

	0.5 Gy	1.5 Gy	3.0 Gy
Chromosome size	*r* = − 0.186	*r* = −0.072	*r* = − 0.783**
Gene density	*r* = −0.475*	*r* = −0.001	*r* = 0.268
Interphase position	*r* = −0.395	*r* = −0.112	*r* = − 0.170

Statistically significant negative correlations are indicated at *p < 0.05 and **p < 0.001

## Discussion

Spontaneously arising CNVs as a source of genetic diversity in human population have been studied extensively [35, 40, 41] and their clinical impact was also demonstrated [42, 43]. Nevertheless, little is known about environmental factors that can induce de novo CNVs. It was shown that de novo CNVs may occur due to influence of replication inhibitors (aphidicolin, hydroxyurea) in vitro in normal human fibroblasts [6, 44]. Earlier we have confirmed these results using mycotoxin aflatoxin B1 as replication inhibitor in cultured human normal leukocytes [8].

Here we demonstrated that laser-driven electron bunches, a direct DNA damaging agent, may induce CNVs in chromosome loci 1p31.1, 7q11.22, 9q21.3, 10q21.1 and 16q23.1 in cultured normal human blood leukocytes. Our data confirmed that hotspots of de novo CNVs mutations defined in normal human fibroblast cell line after ionizing radiation [7] represent also targets for accelerated electrons. Flunkert et al. [45] showed that clones of primary human fibroblasts irradiated with X-ray displayed an increased rate of CNVs in 3p14.2 and 7q11.21. Consistent with this study, our results suggest that locus 7q11.2 is one of the most radiation sensitive sites. We showed that CNVs occurred as duplications or amplifications in all studied chromosome loci which is consistent with results of Arlt et al. [7] where excess of copy number gains over losses was detected. We found only minor differences in the sensitivity of studied sites to radiation. Only locus 1p31.1 was significantly more resistant to radiation at 3.0 Gy compared with other chromosome loci. Nevertheless, the analysis of CNVs in our work is limited by cytogenetically visible changes. We do not exclude the possibility of occurrence of small deletions and duplications as well as more inter-locus differences that are not recognizable by the method applied.

Regarding the mechanisms of ionizing radiation-induced de novo CNVs formation, Arlt et al. [7] hypothesized that the prevalence of copy number gains is difficult to explain via non-homologous end joining (NHEJ) of DNA double strand breaks. Thus, it was suggested that irradiation-induced CNVs are more likely to occur via replication-dependent mechanisms, e.g. repair of more abundant DNA single strand breaks or base lesions. Gains of CNVs in five chromosome loci are also found after irradiation with accelerated electrons; thus, this mechanism is the most appropriate for explaining the observed effects.

According to this analysis of de novo CNVs in human chromosomes 1, 7, 9, 10 and 16 it was shown that the level of fluorescence intensities in CNVs sites negatively correlated with chromosome size after exposure to 3.0 Gy, while no correlation was observed at lower doses of irradiation. These results allow assuming that LDEA-induced CNVs are more probable to occur in small chromosomes rather than in big chromosomes. Earlier Sommer et al. [46], assessing the radiation sensitivity of chromosomes 2, 8 and 14, demonstrated inverse correlation between chromosome DNA content and number of irradiation induced aberrations. Although this contradicts results of Cigarrán et al. [47] who demonstrated, with the exception of chromosome 20, a positive correlation between the DNA content and the number of exchange-type aberrations and the number of breaks in irradiated human blood lymphocytes. No hotspots of de novo CNVs in the chromosomes were identified in offspring of accidentally irradiated parents, confirming that induced CVNs fit the random-effect model of radiation exposure [10]. The literature data thus show both random and non-random distribution of damage across the genome obtained under various irradiation conditions using different biomarkers of radiation.

Negative correlation between increase of fluorescence intensity and gene density in cells irradiated with 0.5 Gy allows to assume that CNVs are more probable to occur in chromosomes with low gene density rather that in chromosomes with high gene density. This is in agreement with results of Rapp et al. [48] research, where the authors with implementation of comet-FISH technique demonstrated that UV-A-induced fragments of gene-rich chromosome 1 can be found in only 3% whereas fragmentation of the gene-poor chromosome 8 was observed in 25% of all comets. Interestingly, Surrallés et al. [49] demonstrated that chromosomes with high gene density are preferentially repaired after influence of mutagen compared to gene-poor chromosomes. Given the limitations of our research, we can only assume that the probability of formation of LDEA-induced mutations in the CNVs sites is inversely related to the chromosomes size and gene density. Further extended studies are needed to describe the distribution of de novo CNVs in chromosomes after irradiation with accelerated electrons.

CNVs observed in current study were persistent in blood cells 72 h post-exposure with accelerated electrons. According to Arlt et al. [7] CNVs can persist in fibroblast cell line after ionizing radiation for more than 7 days. Clones of human primary fibroblast after ionizing radiation displayed an increased rate of CNVs after 20 population doublings [45]. Long-term presence indicates the stability of de novo induced CNVs and their importance as bioindicator of radiation. Analysis of persistent cytogenetic changes in human population is critical for study of biological consequences of radiation exposure [24, 50, 51].

Several previous studies described radiation-induced CNVs in TK6 cells, a human B-cell lymphoblastoid cell line of nonmalignant origin after gamma irradiations with the prevalence of gains [52], and in aneuploid A549 male non-small cell lung cancer adenocarcinoma cell line irradiated with X rays [53]. Increase of CNVs was shown in mouse thymic lymphomas induced by gamma-irradiation in vivo [54]. Current knowledge on DNA copy number alterations in papillary thyroid carcinomas and on strategies to identify radiation-specific changes in these tumors was presented by Zitzelsberger and Unger [55]. According to Arlt et al. [7] the spectrum of DNA damaging agents that lead to increased rates of CNV formation, and the mechanisms by which these agents might act, are very poorly understood.

Thus, research in this area is of particular interest, especially since radiation-induced CNVs can be transmitted to next generation [9, 10] and may play an important role in radiation-induced carcinogenesis [45].

## Conclusions

In summary, we have shown that irradiation with laser-driven electron bunches can induce CNVs in human blood leukocytes in vitro. These CNVs occurred due to duplications or amplifications which according to preliminary data can inversely correlate with chromosome size and gene density. Taking in consideration our previous and current results and literature data we can assume that LDEA may induce replication stress although other mechanisms of CNV induction are not precluded. The observation that CNVs can last in cell population as stable chromosomal changes for several days after radiation exposure allows recommending them for characterization of genetic effects of accelerated electrons. These findings should be complemented with other studies with implementation of more sophisticated methods for CNV analysis.
